# Forme pseudo-condylomateuse d'une nevrodermite de la marge anale

**DOI:** 10.11604/pamj.2014.17.280.2667

**Published:** 2014-04-14

**Authors:** Naoufal Hjira, Rachid Frikh, Abderrahmane ALBouzidi, Mohammed Boui

**Affiliations:** 1 Service de Dermatologie, Hôpital Militaire d'Instruction Mohamed V, Rabat, Maroc; 2 Service d'Anatomie pathologique, Hôpital Militaire d'Instruction Mohamed V, Rabat, Maroc

**Keywords:** Condylome, nevrodermite, marge anale, condyloma, neurodermatitis, anal margin

## Abstract

Cerebral venous thrombosis (CVT) is a rare origin of stroke, the clinical presentation and etiologies vary. The prognosis is shown to be better compared to arterial thrombosis. Magnetic Resonance Imaging (MRI) and MR Venograpgy (MRV) are currently important tools for the diagnostic. We studied 30 cases of CVT diagnosed in the department of neurology of the University Hospital of Fez (Morocco). Patients diagnosed with CVT signs between January 2003 and October 2007 were included in the study. Cerebral CT-scan was performed in 27 cases (90%) while the MRI examination was done in 18 patients (67%); and most patients (90%) received anticoagulant therapy. The mean age of our patients was of 29 years (age range between 18 days and 65 years). A female predominance was observed (70%). The clinical presentation of patients was dominated by: headache in 24 cases (80%), motor and sensory disability in 15 cases (50%), seizures in 10 cases (33%), consciousness disorder in 10 cases (33%). CVT was associated to post-partum in 10 cases (33%), infectious origin in 8 cases (26%), Behcet disease in 2 cases (7%), pulmonary carcinoma in 1 case, thrombocytemia in 1 case and idiopathic in 7 cases (23%). The evolution was good in 20 cases (67%), minor squelaes were observed in 6 patients (20%), while major squelaes was observed in 2 cases. Two cases of death were registered. The CVT is a pathology of good prognosis once the diagnosis is promptly established and early heparin treatment initiated.

## Introduction

L′orientation diagnostique devant des lésions condylomateuses prurigineuses de la marge anale, peut être laborieuse et faire discuter beaucoup d′entités anatomo-cliniques. Il convient que l′enquête étiologique ne retrouve aucune cause, et cela à des proportions variables selon les études, avoisinant les 75% des cas [1]. Le prurit anal essentiel, ou névrodermite péri-anale, ou encore lichen simplex chronicus, est la cause la plus fréquente du prurit anal, mais reste tout de même un diagnostic d′élimination. Nous rapportons le cas d'un homme présentant des lésions pseudo-condylomateuses de la marge anale étiquetés comme névrodermite péri-anale.

## Patient et observation

**Figure 1:**
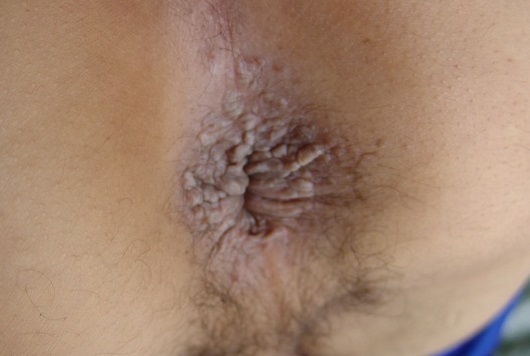
Lésions papulo-nodulaire lichénoïde et pseudo-condylomateuse prenant le pourtour de la marge anale

Un patient de 47 ans, présente depuis plus de 3 ans un prurit anal intense et paroxystique, et a développé des lésions papulo-nodulaires de la marge anale. Le patient a comme antécédents, une diarrhée liquidienne évoluant depuis 4 ans et n′ayant jamais été explorée, ainsi qu′une symptomatologie rhumatologique à type d′arthralgies intermittentes des grosses articulations avec lombalgies. L′examen dermatologique a objectivé de multiples papulo-nodules occupant toute la bordure de la marge anale, ayant tendance à la confluence, tantôt violines lichénoïdes, et tantôt érythémateuses charnues, pseudo-condylomateuses (Figure 1).

**Figure 2:**
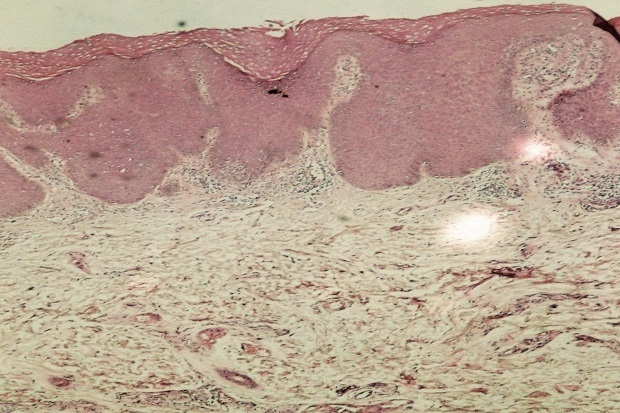
Image histologique d'un revêtement épidermique hyperplasique parakératosique, avec discret infiltrat inflammatoire dermique essentiellement lymphocytaire (HEx200)

L′examen histopathologique n′a objectivé que des remaniements inflammatoires non spécifiques (Figure 2), avec la présence d′une muqueuse de type anale hyperplasique, surmontée par des foyers de parakératose. Le derme sous-jacent est le siège d′un infiltrat inflammatoire essentiellement lymphocytaire. On ne notait pas la présence de koïlocytes, d′infiltrat sous basal lichénien, ou de granulome, et les colorations spéciales à la recherche de dépôts, amyloïdes ou mucineux, sont négatives.

Les explorations morphologiques -anuscopie, rectosigmoïdoscopie et tomodensitométrie abdomino-pelvienne- n′ont pas montré de pathologie proctologique, ou de signes en faveur d′entéropathie inflammatoire chronique. La diarrhée a été mise sur le compte d′une infection parasitaire lors de l′identification de Blastocystis hominis et endolimax nana à l′examen des selles, dont le traitement par métronidazole 1,5 g/j était sans impact sur le prurit anal. Par ailleurs, l′exploration rhumatologique n′a pas objectivé de signes en faveur d'une spondylarthropathie inflammatoire.

Après cette enquête étiologique négative, le diagnostic de névrodermite a été retenu. Le patient fut alors mis sous bétaméthasone dipropionate pommade (traitement supervisé en hospitalier), et un affaissement complet des lésions a été obtenu après 15 jours, ainsi qu′un amendement total du prurit, sans tendance à la récidive lors de la dégression.

## Discussion

L′orientation diagnostique devant des lésions condylomateuses prurigineuses de la marge anale, peut être laborieuse et faire discuter beaucoup d′entités anatomo-cliniques. Ainsi, à côté des classiques localisations péri-anales des dermatoses infectieuses et inflammatoires chroniques [1], comme le lichen plan ou scléro-atrophique, le psoriasis, et les eczémas dans leurs formes atopiques ou de contact, on peut citer la forme condylomateuse de la maladie de Hailey-Hailey [2], l′amylose ano-sacrée [3], ou certaines néoplasies comme la maladie de Bowen ou la maladie de Paget [1]. Les causes proctologiques et digestives [4] sont aussi fréquentes, avec la présence de marisques hémorroïdaires, ou encore une forme pseudo-condylomateuse péri-anale, avec ou sans fistule, d′une maladie de Crohn. Il convient que l′enquête étiologique ne retrouve aucune cause, et cela à des proportions variables selon les études, avoisinant les 75% des cas [1].

Le prurit anal essentiel, ou névrodermite péri-anale, ou encore lichen simplex chronicus, est la cause la plus fréquente du prurit anal, mais reste tout de même un diagnostic d′élimination. C'est un désordre spongiotique chronique de la peau, caractérisé par un prurit ininterrompu, avec une desquamation conséquente.

Elle peut être subdivisée en deux groupes, selon le substratum pathogénique: une névrodermite de novo, qui survient sur un tégument jusque là indemne de toute lésion dermatologique avec des facteurs déclenchants d′ordre psychique et environnemental, et une forme secondaire, marquant le cours évolutif de certaines dermatoses, comme le psoriasis, le lichen scléreux, les dermatophyties des plis, et les infections à HPV (human papilloma virus). Il est à noter que la séquence prurit-grattage marque l′évolution des 2 formes cliniques, et pérennise les lésions cutanées [5].

Le prurit est le signe fonctionnel majeur que les patients qualifient de sévère, rebelle, paroxystique, très gênant, et ne peut être contrôlé malgré les égratignures causées par les ongles. Il est aggravé par la chaleur, la sueur, et le port de vêtements serrés. Les patients nient presque toujours le caractère psychogène de leur prurit, mais reconnaissent souvent son aggravation lors des situations de stress et d′anxiété.

Cliniquement, la névrodermite se manifeste par une ou plusieurs plaques, érythémato-squameuses, excoriées à des degrés variables, et lichénifiées [6]. Ces plaques présentent généralement des troubles leuco-mélanodermiques selon leur ancienneté. La particularité de la région ano-génitale, souvent humide et moite, est la souplesse et la leucoplasie des lésions de névrodermite [5, 6].

Le traitement de la névrodermite se base sur 3 axes. D′abord la restauration de la fonction barrière, moyennant des bains de siège antiseptiques et des émollients, puis la lutte contre l′inflammation chronique par des topiques corticostéroïdes de classe forte, ou des infiltrations intralésionnelles, par du triamcinolone (3-5 mg/séance). Enfin, la prescription d′antihistaminiques sédatifs, en l′absence de contre indications, ou d′antidépresseurs tricycliques, permet de maîtriser la composante nocturne du grattage visant à briser la séquence prurit-grattage omniprésente chez ces patients.

Des traitements topiques à base d′aspirine dissous dans du dichlorométhane [7], ou l'infiltration sous-cutanée de la région anale par du bleu de méthylène à 0.5% [1], ou encore la capsaïcine en pommade à 0.006% [4], semblent être bénéfiques dans cette indication.

## Conclusion

La névrodermite péri-anale est la cause la plus fréquente du prurit anal, c'est un diagnostic d’élimination. Une enquête étiologique appropriée permet de retenir le diagnostic comme le cas de notre patient, permettant ainsi d'adopter les mesures thérapeutiques adéquates.
